# The Role of Pharmacogenetic Biomarkers in Pain

**DOI:** 10.3390/biomedicines13081935

**Published:** 2025-08-08

**Authors:** Ivan Martin da Silva, Adrián Plaza-Díaz, Jesus Ruiz-Ramos, Ana Juanes-Borrego, Pau Riera

**Affiliations:** 1 Pharmacy Department, Hospital de la Santa Creu i Sant Pau, 08025 Barcelona, Spain; imartind@santpau.cat (I.M.d.S.); aplaza@santpau.cat (A.P.-D.); jruizr@santpau.cat (J.R.-R.); ajuanes@santpau.cat (A.J.-B.); 2 Institut de Recerca Sant Pau (IR SANTPAU), 08041 Barcelona, Spain; 3 CIBER de Enfermedades Raras (CIBERER), Instituto de Salud Carlos III, 28029 Madrid, Spain

**Keywords:** pharmacogenetics, pain, opioids, NSAIDs, tricyclic antidepressants

## Abstract

**Background/Objectives**: Pain—whether acute, chronic, or neuropathic—remains a leading cause of disability and reduced quality of life worldwide. Despite advances in pharmacologic options, interindividual variability in response and susceptibility to adverse effects continues to challenge clinicians. In recent years, pharmacogenetics has emerged as a promising approach to optimize analgesic selection and dosing based on patient-specific genetic profiles. This perspective examines current pharmacogenetic evidence in pain management, focusing on validated biomarkers and their clinical implications. **Methods**: A narrative review was conducted of recent literature addressing the impact of genetic polymorphisms on the pharmacokinetics and pharmacodynamics of analgesic agents. Particular focus was given to genes involved in drug metabolism and transport as well as receptor signaling, along with the clinical applications of genotype-informed prescribing. **Results**: Substantial evidence indicates that genetic variants significantly influence patient responses to analgesics, contributing to both inadequate pain relief and heightened sensitivity to adverse effects. The main pharmacogenetic biomarkers appear to be *CYP2C9* (for NSAIDs), *CYP2D6* (for opioids and tricyclic antidepressants), *CYP2C19* (for tricyclic antidepressants) and HLA-B*15:02 and HLA-A*31:01 for carbamazepine. PGx-informed strategies have shown promise in improving analgesic effectiveness, reducing opioid-related complications, and supporting opioid-sparing protocols. **Conclusions**: Pharmacogenetic screening represents a valuable tool for personalizing pain management. Incorporating validated pharmacogenetic biomarkers into clinical practice could improve treatment outcomes and patient safety. Further research, infrastructure development, and clinician education are essential for scaling PGx implementation in pain care.

## 1. Introduction

Pain management remains a complex and unsolved issue in clinical care. Despite significant advancements in both pharmacology and medical technology, post-operative pain is reported by most patients. Approximately 65% of individuals experience moderate to severe pain after surgery, underscoring the limitations of current approaches [1].

Pain is a complex, unpleasant sensory and emotional experience, typically arising from tissue injury. When it is inadequately managed, pain can disrupt vital body functions and impair recovery. It may also contribute to several complications, including pulmonary, cardiovascular, renal dysfunction, gastrointestinal disturbances, infections, and delayed wound healing. Individual responses to analgesics vary widely, adding another layer of complexity to effective pain control. Moreover, patients are frequently prescribed multiple analgesics with the intention of achieving a synergistic therapeutic effect (e.g., paracetamol combined with ibuprofen, or tramadol with nonsteroidal anti-inflammatory drugs). However, these combinations may also increase the risk of adverse effects.

Pharmacogenetics, a cornerstone of personalized medicine, offers a promising strategy to enhance pain management by enabling clinicians to anticipate a patient’s response to analgesics. This approach involves analyzing genetic polymorphisms that influence drug response [2]. Genes related to drug-metabolizing enzymes, receptors, and other components of pain pathways are key determinants of treatment outcomes.

**Figure 1:**
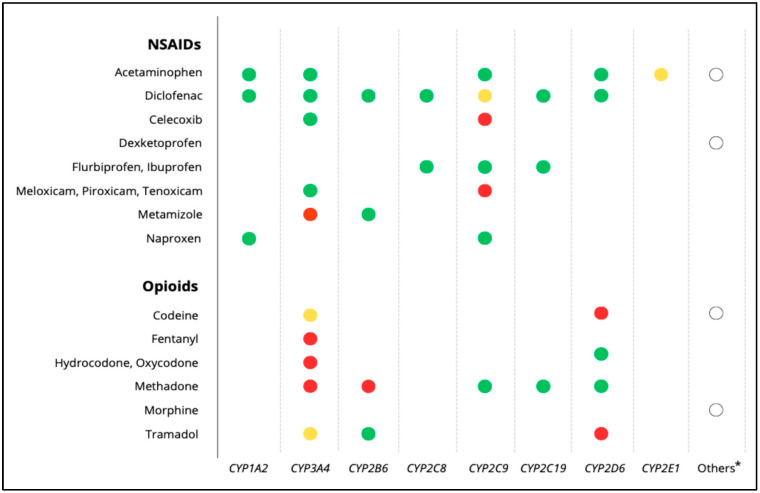
Main metabolic pathways of analgesics, including NSAIDs and opioids. Red dots: major metabolic pathway; yellow dots: major pathway only in the presence of specific inducers or inhibitors; green dots: minor metabolic pathways; white dots *: refers to non-CYP metabolic routes or additional enzymes involved in the metabolism of the respective drugs (e.g., conjugation pathways, esterases, aldehyde dehydrogenase). Source data drug labels and UpToDate [Accessed: July 2025].

Most drugs, including many analgesics, are metabolized by cytochrome P450 (CYP450) enzymes (Figure 1). Polymorphisms in CYP genes can significantly affect enzyme activity and, consequently, treatment outcomes. In addition to genetic factors, variables such as age, body weight, physiological status, sociocultural influences, and drug–drug interactions, also modulate individual drug responses [3].

Integrating pharmacogenetics into perioperative care and broader pain management strategies holds significant potential. This approach can improve therapeutic outcomes, reduce opioid requirements, and minimize adverse effects. By leveraging pharmacogenetic biomarkers to guide drug selection and dosing, clinicians can move beyond the traditional trial-and-error paradigm toward a more precise, individualized model of care.

In this context, we review the current landscape of pharmacogenetic biomarkers most relevant to pain modulation and analgesic response. Our aim is to highlight how emerging genetic insights can inform safer, more effective, and more personalized pain management strategies.

## 2. Pharmacogenetic Biomarkers in Analgesia

### 2.1. Non-Steroidal Anti-Inflammatory Drugs (NSAIDs)

Non-steroidal anti-inflammatory drugs (NSAIDs) are among the most widely prescribed medications for the treatment of acute and chronic pain [4]. They exert their analgesic, anti-inflammatory, and antipyretic effects primarily by inhibiting cyclooxygenase (COX) enzymes—COX-1 and COX-2—thereby reducing the synthesis of prostaglandins involved in pain signaling, inflammation, and fever. While COX-1 inhibition is associated with gastrointestinal (GI) and renal adverse effects, COX-2-selective inhibitors were developed to reduce these risks, although cardiovascular complications have emerged as a concern [4,5]. NSAIDs are a cornerstone in the management of nociceptive and inflammatory pain, such as musculoskeletal disorders, post-operative pain, and cancer-related pain.

Despite their widespread use, NSAIDs exhibit considerable interindividual variability in pharmacokinetics and toxicity profiles [5]. Genetic polymorphisms in metabolic enzymes play a significant role in modulating systemic exposure and the associated risk of adverse outcomes.

Pharmacogenetic Biomarkers and Clinical Implications

*CYP2C9* gene

The cytochrome P450 enzyme CYP2C9 is a major hepatic enzyme responsible for the metabolism of several nonsteroidal anti-inflammatory drugs (NSAIDs), including celecoxib, piroxicam, ibuprofen, meloxicam, and diclofenac [6]. Genetic polymorphisms in the *CYP2C9* gene significantly affect enzymatic activity and clearance, especially in carriers of the *2 (p.R144C; rs1799853) and *3 (p.I359L; rs1057910) alleles [6,7]. These variants are associated with decreased (CYP2C9*2) or no function (CYP2C9*3) and are commonly found in European populations.

Based on the combination of inherited alleles, individuals are classified as poor metabolizers (PMs, activity score 0–0.5), intermediate metabolizers (IMs, score 1–1.5), or normal metabolizers (NMs, score 2) [8]. PMs and IMs exhibit reduced clearance and increased plasma concentrations of NSAIDs, leading to greater risk of gastrointestinal bleeding, renal impairment, and cardiovascular events, particularly with long-term use [9,10].

Evidence from the CPIC dosing guideline underscores that *CYP2C9* polymorphisms markedly influence NSAID pharmacokinetics, with IMs (AS 1.5) able to initiate standard doses of celecoxib, flurbiprofen, ibuprofen, and lornoxicam without significantly elevated exposure, while those with AS 1.0 should start at the lowest dose and closely monitor for adverse effects [9]. PMs (AS 0) should begin therapy at just 25–50% of the lowest recommended dose—or preferably use alternative analgesics not reliant on CYP2C9 (e.g., aspirin, naproxen, metamizole)—and delay any dose increase until steady state is achieved due to prolonged half-life. Regarding meloxicam, its longer half-life (15–20 h) means IMs with AS 1.5 can use standard therapy sparingly while IMs with AS 1 should either start at half the lowest dose or choose an alternative, with dose escalation only after ≥ 7 days; PMs should avoid it entirely due to expected half-lives >100 h [11]. For very long–acting agents like piroxicam and tenoxicam (30–86 h and ~60 h respectively), IMs (AS 1) and PMs (AS 0) are best managed with alternative NSAIDs, given the impracticality of safe titration [6].

Importantly, the clinical impact of *CYP2C9* variants may differ depending on the specific NSAID. Drugs such as aceclofenac, diclofenac, indomethacin, lumiracoxib, metamizole, nabumetone, and naproxen, which are metabolized via multiple pathways, may be less affected by the CYP2C9 phenotype [5,12]. However, in patients requiring chronic NSAID therapy—such as for osteoarthritis or cancer-related pain—genotype-guided selection may offer a safer long-term approach.

*CYP2C8* gene

*CYP2C8*, a gene located in close proximity to *CYP2C9* on chromosome 10, shares overlapping substrate specificity. Interestingly, CYP2C8*3 is in strong linkage disequilibrium with CYP2C9*2. Although CYP2C8’s contribution to NSAID metabolism is less well-defined, it may be relevant in cases involving piroxicam [13] or diclofenac. Evidence is insufficient, however, for the *CYP2C8* genotype to influence clinical decisions [5,14].

Other Pharmacogenes: *UGTs, ABCB1,* and *SLCO1B1*

Likewise, phase II enzymes and transporters such as the UGTs family, ABCB1, and SLCO1B1 have been investigated for their roles in NSAID metabolism and disposition. UGT2B7 contributes to diclofenac glucuronidation, and polymorphisms may influence hepatic toxicity, though data are conflicting [14,15]. *ABCB1* and *SLCO1B1* variants could affect hepatic uptake and efflux, altering drug exposure [5,12]. Still, none of these genes currently meets the threshold for CPIC or DPWG recommendations.

Summary of Evidence

Among NSAIDs, the pharmacogenetic impact of *CYP2C9* is the most clinically relevant and best supported by current evidence. According to CPIC guidelines, reduced CYP2C9 function warrants either dose adjustment or therapeutic substitution to avoid toxicity. While other metabolic and transporter genes may contribute to interindividual variability, their clinical utility remains unvalidated. In patients requiring chronic NSAID therapy or those at higher baseline risk (elderly, renal impairment, cardiovascular comorbidity), pre-emptive *CYP2C9* genotyping represents a pragmatic step toward safer, personalized analgesic care [5,6].

### 2.2. Opioids

Opioids act primarily through activation of the μ-opioid receptor (encoded by the *OPRM1* gene), resulting in inhibition of ascending nociceptive pathways and modulation of pain perception at both spinal and supraspinal levels. They are widely used for the treatment of moderate to severe acute and chronic pain, including post-operative pain, cancer-related pain, and neuropathic pain refractory to other treatments [16].

Although opioids are highly effective analgesics, their use is limited by a narrow therapeutic window, interindividual variability in response, and risk of serious adverse effects such as respiratory depression, sedation, dependence, and opioid-induced hyperalgesia [17]. Pharmacogenetic variability, especially in genes encoding drug-metabolizing enzymes, receptors, and transporters, contributes to this variability.

Pharmacogenetic Biomarkers and Clinical Implications

*CYP2D6* gene

CYP2D6 is a highly polymorphic enzyme that plays a clinically relevant pharmacogenetic determinant in opioid therapy, particularly for drugs that require metabolic activation (e.g., codeine and tramadol) that must be converted by CYP2D6 to morphine and O-desmethyltramadol, respectively, to exert meaningful analgesic effects. Over 130 allelic variants have been described [18], leading to substantial differences in enzyme activity and to four major phenotypes: PM, IM, NM, and UM [19].

PMs carry two non-functional alleles (*3, *4, *5, *6) and exhibit severely impaired conversion of prodrugs, resulting in therapeutic failure. At the opposite end, ultrarapid metabolizers often carry multiple active gene copies (*1xN, *2xN), leading to excessive generation of active metabolites and an increased risk of opioid-induced toxicity, including potentially life-threatening respiratory depression [19,20]. IMs commonly carry one non-functional allele and one reduced-function allele (*9, *10, *17, *29, *41), or two reduced-function alleles, resulting in decreased enzymatic activity and potentially diminished efficacy. NMs typically carry two fully functional alleles (*1, *2) or one functional plus one reduced-function allele, and generally respond as expected to standard doses [20,21].

These pharmacogenetic differences have critical clinical implications. Both CPIC and DPWG guidelines advise against the use of codeine and tramadol in individuals classified as CYP2D6 PMs or UMs, due to a significantly increased risk of therapeutic failure or opioid toxicity, respectively. In PMs, the markedly reduced or absent formation of active metabolites such as morphine or O-desmethyltramadol results in suboptimal analgesic response, while in UMs, accelerated conversion leads to supratherapeutic levels and heightened risk of adverse events, including life-threatening respiratory depression. In both cases, an alternative analgesic not metabolized (or minimally metabolized) by CYP2D6 should be used. For IMs, both guidelines recommend close monitoring of analgesic response, with the DPWG further suggesting dose escalation or substitution if efficacy is insufficient. Notably, the DPWG discourages any dose adjustments in UMs or PMs, emphasizing instead the need to completely avoid these drugs in such patients due to the unpredictability of their clinical effects [20,22].

For opioids such as hydrocodone and oxycodone, which are only partially metabolized by CYP2D6, the clinical evidence linking genotype to analgesic response is less consistent. The CPIC acknowledges that *CYP2D6* polymorphisms may affect the formation of active metabolites—hydromorphone and oxymorphone, respectively—but concludes that current data are insufficient to support genotype-guided prescribing for these drugs. Similarly, the DPWG notes a potential reduction in efficacy among poor metabolizers, particularly with oxycodone; however, no specific action is recommended unless pain control proves inadequate. In such cases, switching to an opioid not reliant on CYP2D6 metabolism may be considered. Overall, both guidelines assign a lower clinical relevance to pharmacogenetic testing for these agents compared to codeine or tramadol, and routine implementation is not advised [17,20,22].

*OPRM1* gene

The μ-opioid receptor, encoded by the *OPRM1* gene, is the principal site of action for most opioids. The A118G polymorphism (rs1799971), which results in an asparagine-to-aspartate substitution at position 40 (N40D), has been associated with altered receptor expression and binding affinity [21,23]. Carriers of the G allele have been reported to require higher opioid doses to achieve equivalent analgesia, potentially due to reduced receptor sensitivity or altered downstream signaling [24,25].

Despite its biological plausibility and a large body of observational studies, the clinical impact of *OPRM1* genotyping remains uncertain. The effect size is modest, the findings are inconsistent across ethnic groups and pain conditions, and most studies do not adjust for concomitant pharmacokinetic variation. As a result, CPIC does not consider *OPRM1* genotype actionable [20,22]. It may still contribute to interindividual variability in opioid response as part of polygenic or multivariate models, but its utility in isolation is limited.

COMT gene

Catechol-O-methyltransferase (COMT) regulates the degradation of catecholamines such as dopamine and norepinephrine in the central nervous system, both of which play roles in endogenous pain modulation. Numerous SNPs have been shown to affect enzymatic activity and modulate an individual’s sensitivity to pain [23].

The most extensively studied functional polymorphism, Val158Met (rs4680), is associated with reduced enzymatic activity in Met allele carriers, particularly in the Met/Met genotype. These individuals tend to have higher catecholaminergic tone and may show greater pain sensitivity and altered response to opioids [26,27]. Evidence suggests that Met/Met individuals may respond better to opioids—or conversely, be more prone to opioid-induced hyperalgesia—but findings are not consistent [28].

Three major *COMT* haplotypes—low (LPS), average (APS), and high pain sensitivity (HPS)—have been linked to experimental pain sensitivity. Defined by combinations of SNPs (rs6269, rs4633, rs4818, rs4680), these haplotypes (LPS: GCGG; APS: ATCA; HPS: ACCG) are associated with interindividual variability in post-operative opioid requirements [23]. COMT’s effect is likely context-dependent and influenced by environmental and psychological factors. 

Currently, CPIC does not support the use of *COMT* genotyping in clinical decision-making for opioid therapy. Like OPRM1, COMT may become relevant in integrative pharmacogenomic models, but not as a standalone biomarker [20,22].

Other Pharmacogenes: *CYP3A*, *CYP2B6*, *ABCB1*, *UGT2B7*

Other genes involved in opioid pharmacokinetics have been studied. CYP3A enzymes are responsible for the N-dealkylation and inactivation of synthetic phenylpiperidines such as fentanyl and alfentanil, as well as semisynthetic opioids like hydrocodone and oxycodone. Methadone is primarily metabolized through N-demethylation by CYP2B6 [20,29]. While genetic variability in these enzymes may influence drug clearance and systemic exposure, findings have been inconsistent, and environmental factors—such as the presence of enzyme inducers or inhibitors—tend to have a greater influence. As a result, these genetic variants have not reached the level of evidence required for clinical implementation and their determination is not recommended in current CPIC guidelines [29].

The efflux transporter ABCB1, also known as the multidrug resistance protein 1 (*MDR1*) gene, which modulates blood–brain barrier permeability of opioids, also has been studied in relation to morphine and methadone CNS exposure [30]. The C3435T polymorphism (rs1045642) has shown variable associations with efficacy and adverse effects, but replication has been poor [31]. Likewise, UGT2B7, which glucuronidates morphine into morphine-3- and 6-glucuronide, has polymorphisms that may influence metabolite ratios and clinical response, though again, evidence is insufficient for routine use [32].

Summary of Evidence

At present, only *CYP2D6* genotyping is supported by strong, converging recommendations from CPIC and DPWG, specifically in relation to codeine and tramadol. Other gene–drug associations are biologically plausible but not yet clinically validated. In practice, *CYP2D6* genotyping may serve as a valuable tool to avoid treatment failure or adverse outcomes in opioid prescribing, particularly in perioperative or primary care contexts where codeine and tramadol remain in use. Broader genetic testing panels, incorporating emerging biomarkers like *CYP3A4*, *OPRM1*, or *COMT*, may enhance predictive power in the future but require further validation through prospective studies.

### 2.3. Antidepressants, Anticonvulsants, and Gabapentinoids

Antidepressants, certain anticonvulsants, and gabapentinoids represent first- and second-line pharmacologic options for the management of neuropathic pain. Tricyclic antidepressants (TCAs) such as amitriptyline and nortriptyline, inhibit the reuptake of serotonin and norepinephrine in the descending inhibitory pathways and have additional sodium channel–blocking and anticholinergic effects [8]. Selective serotonin reuptake inhibitors (SSRIs), including paroxetine, fluoxetine, and sertraline, as well as serotonin-norepinephrine reuptake inhibitors (SNRIs) such as duloxetine and venlafaxine, have also demonstrated utility in pain syndromes like fibromyalgia and diabetic neuropathy. Their analgesic effects are thought to arise from enhanced monoaminergic modulation of central nociceptive pathways. Among them, duloxetine has the strongest evidence base in pain contexts and is approved for several chronic pain indications [5].

Anticonvulsants such as carbamazepine and oxcarbazepine stabilize hyperexcitable neuronal membranes via sodium channel inhibition [20], while gabapentinoids (gabapentin and pregabalin) bind the α2δ subunit of voltage-gated calcium channels, reducing excitatory neurotransmitter release.

Although widely prescribed, these agents exhibit marked interindividual variability in efficacy and tolerability. Their narrow therapeutic index and side-effect profile—particularly for TCAs and carbamazepine—make them strong candidates for pharmacogenetic optimization.

Pharmacogenetic Biomarkers and Clinical Implications

#### 2.3.1. Tricyclic Antidepressants (TCAs)

*CYP2D6* gene

Amitriptyline and nortriptyline are extensively metabolized by CYP2D6 to their less active hydroxylated forms [8]. Genetic polymorphisms in *CYP2D6* affect plasma concentrations, half-life, and side-effect burden. Poor metabolizers (PMs) are at increased risk of drug accumulation and adverse effects such as sedation, anticholinergic burden, and QT prolongation, whereas ultrarapid metabolizers (UMs) may experience subtherapeutic levels and reduced efficacy.

CPIC guidelines recommend a 50% dose reduction in CYP2D6 PMs and the use of alternative agents in UMs due to potential treatment failure [33]. DPWG expands on this, offering more granular dosing guidance. For CYP2D6 intermediate metabolizers (IMs), a 30% dose reduction is not necessary, but a reduced dose—approximately 70% of the standard—is recommended, alongside monitoring for adverse effects and plasma concentrations of imipramine and desipramine. For CYP2D6 PMs, a reduction to 30% of the standard dose is advised with similar monitoring, given the high risk of toxicity. In CYP2D6 UMs, a dose increase of up to 1.7 times the standard dose may be considered if hydroxy metabolite–induced cardiotoxicity is not expected; otherwise, imipramine should be avoided altogether [34].

This pharmacogenetic guidance is particularly relevant in pain settings, where TCAs are typically administered at lower doses. Nonetheless, genetic variability can still lead to significant interindividual differences in tolerability and efficacy.

*CYP2C19* gene

CYP2C19 contributes to the demethylation of amitriptyline to nortriptyline and of imipramine to desipramine, as well as their further metabolism. PMs may exhibit increased exposure to both parent and active metabolites, raising the risk of adverse effects.

According to CPIC, dose reduction or alternative therapy should be considered when both CYP2D6 and CYP2C19 pathways are impaired. DPWG recommends that CYP2C19 PMs take 70% of the standard dose of imipramine or desipramine, accompanied by close monitoring of side effects or plasma concentrations to guide further dose adjustments. Alternatively, avoidance of imipramine is suggested in high-risk cases. No dose adjustments are recommended for CYP2C19 UMs or IMs [33,34]. In all cases, TDM is encouraged, aiming for a combined plasma concentration of imipramine and desipramine.

*ABCB1* gene

The efflux transporter P-glycoprotein, encoded by ABCB1, modulates blood–brain barrier penetration of various TCAs. Polymorphisms such as C3435T (rs1045642) have been studied in association with CNS exposure and antidepressant efficacy [31]. Some evidence suggests altered distribution and therapeutic response in carriers of certain variants, but results are inconsistent.

Currently, neither CPIC nor DPWG includes ABCB1 in their formal guidance for TCAs. Its clinical utility remains investigational and likely context dependent.

#### 2.3.2. Anticonvulsants: Carbamazepine and Oxcarbazepine

HLA-A*31:01 and HLA-B*15:02 genes

Carbamazepine, widely used for trigeminal neuralgia and other neuropathic syndromes, poses a significant risk of severe cutaneous adverse drug reactions in genetically susceptible individuals [33]. Among these, Stevens–Johnson syndrome (SJS) and toxic epidermal necrolysis (TEN) are the most serious. The presence of HLA-B*15:02 is strongly associated with these reactions, particularly in individuals of East Asian descent. Additionally, HLA-A*31:01 has been linked to a broader spectrum of hypersensitivity reactions, including drug reaction with eosinophilia and systemic symptoms (DRESS) and maculopapular eruptions, especially in populations of European and Japanese ancestry [17,35].

The CPIC guidelines recommend genotyping for HLA-B*15:02 before initiating carbamazepine in patients of at-risk ancestry. If the allele is detected, carbamazepine should be avoided. For carriers of HLA-A*31:01, CPIC suggests considering alternative therapies due to the elevated risk of various hypersensitivity reactions, though the recommendation is less stringent than for HLA-B*15:02 [36]. DPWG classifies HLA-B*15:02, HLA-B*15:11, and HLA-A*31:01 as clinically actionable markers for carbamazepine hypersensitivity. For individuals carrying any of these alleles, the DPWG recommends avoiding carbamazepine. If no suitable alternative is available, patients must be closely monitored and explicitly instructed to report any rash or signs of hypersensitivity immediately. Carbamazepine is contraindicated in HLA-B*15:02-positive individuals [37].

Oxcarbazepine, a structural analogue of carbamazepine frequently used in similar pain contexts, carries a comparable but generally lower risk of cADRs. However, HLA-B*15:02 has also been associated with an increased risk of SJS/TEN in response to oxcarbazepine, particularly in Asian populations. As such, both CPIC and DPWG recommend HLA-B*15:02 genotyping prior to initiating oxcarbazepine in patients of relevant ancestry. In confirmed carriers, oxcarbazepine should be avoided, and alternative agents should be considered. In contrast, HLA-A*31:01 has not been definitively associated with oxcarbazepine-induced hypersensitivity, and thus is not currently included in clinical recommendations for this drug [36,37].

Incorporating pharmacogenetic screening into routine prescribing pain management offers a pragmatic strategy to enhance patient safety and reduce the incidence of severe adverse drug reactions.

#### 2.3.3. Selective Serotonin and Norepinephrine Reuptake Inhibitors (SNRIs) and Selective Serotonin Reuptake Inhibitors (SSRIs)

*CYP2D6* and *CYP2C19* genes

Selective serotonin reuptake inhibitors (SSRIs) and serotonin-norepinephrine reuptake inhibitors (SNRIs) are commonly used for both psychiatric and pain-related indications, including fibromyalgia and neuropathic pain [38]. Genetic variability in *CYP2C19* and *CYP2D6* significantly influences the pharmacokinetics and clinical response to SSRIs and SNRIs, particularly in the context of pain management.

CYP2C19 plays a major role in the metabolism of citalopram, escitalopram, and sertraline. PMs may experience increased plasma concentrations and a higher risk of dose-related adverse effects such as QT prolongation (notably with citalopram), while UMs are at risk of reduced efficacy due to low systemic exposure [39].

CPIC recommends dose reduction or alternative therapy in CYP2C19 PMs and avoidance of these agents in UMs. For sertraline, a lower starting dose is recommended in PMs, with no change needed for other phenotypes unless treatment response is inadequate.

CYP2D6 is primarily involved in the metabolism of paroxetine and fluoxetine. PMs may show elevated drug levels and increased risk of adverse events, while UMs may not achieve therapeutic plasma concentrations. CPIC recommends considering alternative agents or genotype-informed dose adjustments in these cases [40,41].

Duloxetine, though metabolized by both CYP1A2 and CYP2D6, is not included in CPIC’s formal recommendations due to limited evidence. Empirical titration is advised. DPWG’s guideline likewise does not consider the CYP2D6–duloxetine interaction clinically actionable and does not recommend genotype-based dose modifications. For venlafaxine, however, DPWG classifies the CYP2D6 interaction as significant. IMs and PMs are at risk of toxicity and suboptimal response; avoidance is advised. If used, a dose reduction and close monitoring of plasma levels and side effects are recommended. No dose adjustment is necessary in UMs [41].

Other Pharmacogenes: *ABCB1*, *SLC6A4*, and *HTR2A*

*ABCB1*, *SLC6A4*, and *HTR2A* polymorphisms have been investigated for their potential role in SSRI response and tolerability (e.g., blood–brain barrier transport, serotonin transporter activity, receptor sensitivity), CPIC guidelines do not currently include them in clinical recommendations. Results across studies have been inconsistent, and no actionable guidance has been issued for their use in guiding SSRI/SNRI therapy.

#### 2.3.4. Gabapentinoids: Gabapentin and Pregabalin

Gabapentinoids are not substrates for Cytochrome P450 enzymes and are excreted unchanged via the kidneys. Their pharmacokinetics are generally predictable and unaffected by metabolic genetic variation. To date, no pharmacogenetic marker has demonstrated clinical utility in guiding the use of gabapentin or pregabalin.

Although some exploratory studies have investigated polymorphisms in *GABRA1* gene (gamma-aminobutyric acid receptor) and L-type amino acid transporter 1, encoded by the *SLC7A5* gene, and their influence on analgesic response, evidence is preliminary and inconsistent [42,43].

Neither CPIC nor DPWG provide pharmacogenetic recommendations for these agents, and they remain preferred options in patients where gene–drug interactions are a concern.

Summary of Evidence

Among adjuvant analgesics for neuropathic pain, tricyclic antidepressants, serotonin reuptake inhibitors, and carbamazepine stand out for their well-established pharmacogenetic considerations. *CYP2D6* and *CYP2C19* genotyping can inform safe and effective use of amitriptyline and nortriptyline, as well as SSRIs like paroxetine and escitalopram, and SNRIs such as venlafaxine, for which metabolizer status significantly influences drug exposure and tolerability. HLA screening is critical for avoiding life-threatening hypersensitivity to carbamazepine. In contrast, gabapentinoids currently lack actionable PGx associations but serve as a reliable alternative in genetically at-risk patients. Integrating these pharmacogenetic principles into analgesic selection can enhance therapeutic precision and minimize harm in patients with chronic or neuropathic pain syndromes.

In conclusion, several biomarkers have been associated with response/toxicity to analgesics. The main biomarkers along with their clinical relevance are summarized in Table 1. They are classified according to the following evidence levels: Level 1A = dose adjustment recommended by both CPIC and DPWG; Level 1B = recommendation from CPIC or DPWG; Level 2 = mentioned but not actionable; Level 3 = no recommendation from CPIC or DPWG.

## 3. Clinical Implications and Future Perspectives

### 3.1. From Single-Gene Testing to Multi-Omic Pain Signatures

Evidence accumulated in the past three years points toward diminishing returns from looking at *CYP2D6* or *CYP2C9* in isolation. New integrative models that combine rare + common variants across metabolism (e.g., *CYP2D6/2C9/3A4*), transport (*ABCB1*), receptor pharmacodynamics (*OPRM1*), and modulators of pain circuitry (*COMT*, ion-channel genes) are beginning to outperform single-gene tests in predicting both analgesic response and baseline pain sensitivity. Large prospective cohorts that layer genomics with epigenetics, serum proteomics, and quantitative sensory testing are now underway and are likely to yield clinically actionable “pain-omics” panels within the next five years [23].

### 3.2. Implementation Science and Real-World Pragmatism

The field is pivoting from proof-of-concept RCTs to large, pragmatically embedded trials that answer whether genotype-guided prescribing improves outcomes in busy peri-operative and primary care workflows. The IGNITE network’s ADOPT-PGx program (acute and chronic pain arms) and the PGx-ACT trial are deliberately testing scalable models such as delayed-testing controls, virtual consent, and point-of-care reporting, creating blueprints for future health system rollouts [44].

### 3.3. Clinical Decision Support (CDS) That Clinicians Will Use

Early PGx CDS often surfaced from static PDF reports; new iterations integrate star-allele calls directly into the electronic health record with interruptive alerts, G-standard dosing logic, and links to CPIC tables. Recent reviews of PGx CDS show that embedding genotype calls at medication-order entry is associated with higher guideline concordance and faster prescribing decisions, paving the way for “zero-click” PGx at the bedside [45].

### 3.4. Equity, Ancestry, and Allele Frequency Gaps

Actionable opioid-metabolizer alleles cluster differently across ancestries (e.g., >40% actionable CYP2D6 variants in multi-ethnic Singapore versus ~8% in Europeans), underscoring the importance of enrolling diverse populations and validating algorithms on non-European genomes. Future trials must stratify by ancestry-specific activity scores and explore copy-number variation and structural alleles that remain under-represented in current panels [46].

### 3.5. Digital Phenotyping and AI-Driven Dosing

Wearable-derived pain trajectories, opioid smart-pill bottles, and NLP extraction of adverse-event mentions are starting to feed machine-learning models that can “phenoconvert” static genotypes into dynamic dose recommendations. Coupling such models with rapid (<4 h) point-of-care genotyping may finally allow peri-operative teams to order a PGx panel in the pre-admission clinic and act on the results in the recovery room [47,48].

### 3.6. Real-World Evidence (RWE) and Health–Economic Data

Completed RCTs are now feeding into payer-facing cost–utility analyses that incorporate opioid-related adverse event avoidance and shortened lengths of stay. Early models indicate that pre-operative PGx testing becomes cost saving when ≥15% of the surgical population carries a clinically actionable genotype [49].

Below is a concise snapshot of the evidence pipeline driving pharmacogenetic-guided pain management. Table 2 collates five influential clinical trials to illustrate how multi-gene testing is being embedded in real-world workflows. Over the next 3–5 years we anticipate the following:

- Pre-emptive panel testing becoming part of routine pre-operative assessments, starting with high-risk surgeries.
- Joint PGx + opioid-sparing protocols (regional anesthesia, NSAID rotation) assessed in factorial designs.
- Cost effectiveness and payer coverage analyses embedded as secondary outcomes to accelerate reimbursement decisions.
- Regulatory alignment as CPIC/DPWG tables converge and updated NSAID and opioid labels incorporate genotype-based dosing ranges.

In conclusion, the field is moving rapidly from “should we test?” to “how do we implement wisely and equitably?”. The next horizon lies in translating these genomic insights into automated, ethically grounded, and globally accessible pain management strategies.

## 4. Conclusions

Pharmacogenetics has emerged as a key pillar in the move toward personalized pain management. The increasing body of evidence supports the clinical utility of genotyping some pharmacogenetic biomarkers such as *CYP2D6*, *CYP2C9*, HLA-B*15:02, or HLA-A*31:01 across a wide range of analgesics. By incorporating genetic information into prescribing decisions, clinicians can mitigate the risks of adverse drug reactions, improve analgesic effectiveness, and reduce the trial-and-error approach that often burdens patients and healthcare systems alike.

Despite notable progress, several challenges persist in the field of pharmacogenetics in pain management. The main limitations for its implementation are the inconsistent clinical relevance of certain polymorphisms, the underrepresentation of specific populations in studies, insufficient bioinformatic resources, and the need for effective integration of decision support tools into clinical practice. Moreover, there are few studies focused on patient-centered outcomes, which hampers the ability to assess its real-world effectiveness and patient benefits. While pharmacogenetics plays a significant role in modulating analgesic response, it is important to acknowledge that an overemphasis on genetic factors could potentially overlook the broader clinical and environmental influences that also shape patient outcomes. Pharmacogenetics should be considered as one of many components in the complex interplay of factors contributing to treatment effectiveness and safety. Additionally, the lack of a quantitative synthesis represents a limitation of our manuscript, as it may constrain the depth of the analysis and the ability to draw more definitive conclusions.

Addressing these gaps requires future research to adopt multimodal, polygenic, and context-aware strategies that combine pharmacogenetics with digital phenotyping, real-world data, and machine learning to develop adaptive models for analgesic optimization. As healthcare systems increasingly implement pre-emptive genotyping, the prospect of safer and more effective pain therapies becomes attainable. Realizing the full potential of pharmacogenetics in pain management will depend on coordinated efforts among researchers, clinicians, and policymakers to overcome current limitations and establish them as a standard component of care.

## Figures and Tables

**Table 1:** Summary of Clinically Relevant Pharmacogenetic Interactions in Pain Management. A comprehensive overview of drug–gene pairs across analgesic classes, including level of evidence and recommendations from CPIC and DPWG.

	Genes	Level of Evidence †	Drug(s)	CPIC Recommendation	**DPWG Recommendation**
NSAIDs	*CYP2C9*	Level 1B	Celecoxib, Flurbiprofen, Ibuprofen, Lornoxicam	PMs (AS 0) should begin therapy at just 25–50% of the lowest recommended dose or use alternative analgesics	No recommendation
		Level 1B	Meloxicam	IMs (AS 1) should either start at half the lowest dose or choose an alternative; PMs should avoid it	No recommendation
		Level 1B	Piroxicam, Tenoxicam	IMs (AS 1) and PMs (AS 0) are best managed with alternative NSAIDs	No recommendation
	*CYP2C8*	Level 2	Ibuprofen, Piroxicam, Diclofenac	Not actionable	No recommendation
	*UGTs*, *ABCB1* and *SLCO1B1*	Level 3	NSAIDs	No recommendation	No recommendation
Opioids	*CYP2D6*	Level 1A	Codeine, Tramadol, Oxycodone, Hydrocodone	Avoid codeine/tramadol in PMs/UMs, and monitor in IM patients	Avoid codeine/tramadol in PMs/UMs
	*OPRM1*	Level 2	Morphine, Hydrocodone, Fentanyl	Not actionable	No recommendation
	*COMT*	Level 2	All opioids	Not actionable	No recommendation
	*CYP3A4/5*	Level 3	Fentanyl, Alfentanil, Oxycodone	No recommendation	No recommendation
	*CYP2B6*	Level 2	Methadone	Not actionable	No recommendation
	*ABCB1* and *UGT2B7*	Level 3	Morphine, Methadone	No recommendation	No recommendation
Anticonvulsants	HLA-B*15:02	Level 1A	Carbamazepine, Oxcarbazepine	Avoid if possible	Carbamazepine is contraindicated
	HLA-A*31:01	Level 1A	Carbamazepine	Considering alternative therapies.	Avoid if possible.
Antidepressants	*CYP2C19*	Level 1A	Amitriptyline, Nortriptyline,	Dose reduction or alternative therapy in UMs/PMs	70% of the standard dose in UMs/PMs patients
		Level 1A	Citalopram, Escitalopram, Sertraline	Dose reduction or alternative therapy in PMs and avoidance of these agents in UMs	In PMs escitalopram/citalopram dose should not exceed 50% of the maximum dose, and it should be avoided in UMs (not citalopram)
	*CYP2D6*	Level 1A	Amitriptyline, Nortriptyline	50% dose reduction in PMs and the use of alternative agents in UMs	30% dose reduction in IMs/PMs, and 1.7 times dose increase in UMs
		Level 1A	Paroxetine, Fluoxetine	Considering alternative agents or genotype-informed dose adjustment	Paroxetine should be avoided in PMs patients
		Level 2	Duloxetine	Not actionable	Not actionable
		Level 1B	Venlafaxine	Not actionable	IMs/PMs are at risk of toxicity and suboptimal response; avoidance is advised
	*SLC6A4*	Level 2	SSRIs/SNRIs	Not actionable	No recommendation
	*HTR2A*	Level 2	SSRIs/SNRIs	Not actionable	No recommendation
Gabapentinoids	*GABRA1*	Level 3	Gabapentin, Pregabalin	No recommendation	No recommendation
	*SLC7A5*	Level 3	Gabapentin, Pregabalin	No recommendation	No recommendation

† Evidence levels: Level 1A = dose adjustment recommended by both CPIC and DPWG; Level 1B = recommendation from CPIC or DPWG; Level 2 = mentioned but not actionable; Level 3 = no recommendation from CPIC or DPWG.

**Table 2:** Ongoing and Completed CT on Pharmacogenetics-Guided Pain Management. Key studies evaluating genotype-guided analgesic prescribing, highlighting design, intervention, and primary outcomes.

Trial/Year	Population and Setting	Genetic Focus and Intervention	Primary Outcome(s)/Status
NCT05452694/2022	235 adults after lumbar fusion or decompression surgery (UPMC, USA)	16-gene opioid/NSAID panel (*CYP2D6*/*3A4*/*2B6*, *OPRM1*, *ABCB1* ± risk score) returned pre-discharge	Composite opioid-related adverse events (sedation, respiratory depression, PONV) to 72 h; recruiting
NCT05525923/2023	200 adults undergoing thoracotomy/VATS lung resection	Same 16-gene panel guiding oxycodone dosing and rescue choices	90-day chronic post-surgical pain; opioid-AE rate; recruiting
NCT04685304/2023 †	315 primary care adults on tramadol/codeine/hydrocodone (PGx-ACT, USA)	Immediate vs. 6 mo-delayed PGx (*CYP2D6* ± *CYP2C19*) + pharmacist CDS	Δ Pain-intensity at 3 mo; active, not recruiting
NCT06669650/2024	208 opioid-naïve adults, mixed surgeries (UTenn, USA)	Rapid saliva panel—CYP2D6 phenotype branches to hydromorphone vs. oxycodone regimen	Persistent opioid use at 90 days; active, not recruiting
NCT01140724/2022	1200 pediatric tonsillectomy patients, multi-center USA	Multi-gene morphine panel (*COMT*, *CYP2D6*, *OPRM1*, *ABCC3*) with opioid-avoidance algorithm	Genotype-morphine dose–response and opioid-AE composite; active, not recruiting

† Trials registered earlier but issued new protocols/major amendments and have generated peer-reviewed updates. Recruitment status per registry updates accessed 15 June 2025.
